# Think Before Chopping a Diabetic Foot: Insight to Vascular Intervention

**DOI:** 10.7759/cureus.1194

**Published:** 2017-04-25

**Authors:** Ahsan Zil-E-Ali, Saadia Shafi, Muhammad Hammad Ali

**Affiliations:** 1 General Surgery, Pak Red Crescent Hospital; 2 Department of Psychiatry, Shifa College of Medicine; 3 Biochemistry, Fatima Memorial Hospital

**Keywords:** vascular surgery, diabetes mellitus, diabetic foot

## Abstract

Diabetes mellitus is a most commonly occurring chronic disease around the world, resulting in damage to multiple organs. One of the consequences of poorly controlled diabetes is vascular damage resulting in peripheral artery disease, leading to inadequate perfusion of the foot and eventually gangrene and amputation. Research over the past decade or so has provided us with the statistics that vascular intervention has better clinical outcomes including patient mortality, morbidity, quality of life, and patient satisfaction. This editorial advocates the importance of pursuing a vascular plan prior to a limb salvaging procedure. We highlight some important aspects of saving a diabetic foot and encourage the importance of giving a vascular trial.

## Editorial

According to available statistics, 422 million people suffer from diabetes mellitus in the world, which makes almost 8.5% of the whole human kind [1]. It’s been a known pathology that the glycosylation of collagen in the vascular walls narrows the lumen and hence leads to a number of complications, one of which is peripheral artery disease and ischemia of the foot. The gangrene formed due to the lack of nutrients eventually leads to soft tissue infections and fatal bone infection, osteomyelitis. 

To reduce the spread of the bone infection, the limb is salvaged, and the patient has to live as an amputee for the rest of his/her life. Usually the foot isn’t trialled with intense therapies and the easier route is to cut the body part. The foot can be saved, and multiple investigations have supported the vascular routing and high dose antibiotics that have saved limbs from osteomyelitis and series of amputations from ray to full limb severance by trivial interventions.

The baseline of treating a diabetic foot stands on two principles: 1) revascularization and establishing a nutrient path through major pedal vessels, 2) reducing the infections by antimicrobial management. The second principle involves the reduction in microbial action that is an adjunct point for vascular surgeons and experts of infectious diseases that can control the local septic milieu and developing osteomyelitis at an early stage. This involves the conventional medical approach and in the past few years the advancement has mainly been in presenting similar pharmacological compounds with varying spectrums and efficacies. Better clinical outcomes have also been observed with a combination of infected bone resection and placement of antibiotic-loaded bone cement [2].

The first principle is focal in nature. The vascular intervention can be the older technique that may involve the bypass or the newer and more promising endovascular approach. Bypasses have been replaced by the expertise of today’s vascular practice and involves lesser side effects and better results. In addition, bypasses may be a better option in wider vessels above the knee, which are usually less related to a diabetic necrotic foot. It has been recorded that most of the occlusive cases of diabetes involves below-the-knee arteries for which open surgery and endovascular surgery have same end point ulcer healing ranging from 78% to 85% and is usually the choice of the surgical team, and in most of the world class institutions, endovascular is the main approach for the diabetic foot [3].

A percutaneous transluminal angioplasty (PCTA) can be the gold standard in approaching narrower vessels with or without stenting, which may improve blood flow and aid healing. Although, revascularization in bedridden patients is still debatable. Apart from the known multiple techniques of vascular intervention versus a limb amputation, the first option offers a much better prognosis, in many ways even a better lifestyle, though the set of available vascular interventions are still comparable and the efficacy of one over the other requires a lot of research and wider scope in this field. Amputation itself causes a higher mortality rate. For now, PCTA with stenting is considered a better option, which may be a biased opinion considering its higher prevalence of choice among the general and vascular surgeons [4]. The vascular approach has emerged as physically and economically superior to amputation but multiple barriers are still present which limits it as standard therapy.

The daily surgery practice with older knowledge has led to thousands of amputations in Pakistan and the room of research has not been filled by innovational ideas. A research paper published in Hungary concluded the long term limb survival and mortality among diabetics and non-diabetic groups. It was recorded that the survival rates of a limb was 65.8% in the diabetic group whereas it was 89.6% in the non-diabetics, while death occurrence recorded was 13.5% versus 6.8% in diabetic and non-diabetic leg subgroups, respectively [5]. This could be one of the reasons for preferring amputation over limb salvage methods in patients with diabetes as procedure failure rates are higher, which ultimately leads to removing the limb. Consequently, in such patients the medical cost is doubled, as well as the physical and psychological burden of going through multiple procedures and in-hospital stay. Nevertheless, cutting a limb is easier than saving it, which obviously requires more knowledge, skills, training and expertise. Since the majority of the population have access to rural health care facilities and since such expertise is present in tertiary care hospitals located in urban locations, the treatment scale is shifted towards performing amputations rather than limb saving procedures. Furthermore, a team of experts comprising a vascular surgeon, internist, interventional radiologist and experienced staff is essential for a successful procedure and recovery.

In our view, we need to consider that a diabetic patient battles through multiple organ damage, routine investigations and the mental misery of a chronic condition that requires years of rigorous medication routine and follow-ups; an amputation is an add-on to the patient’s despair. As physicians and surgeons, the chopping of a limb should be the last option if we have highly competent vascular surgeons who can intervene in the problem when the general surgeon has failed to offer better options to the ailing foot. This will require time, training and interest in this field by budding surgeons. We need to educate health care professionals regarding the emergence of newer procedures which are safer, cost effective and have more chances of improving the quality of life in diabetics. A lot more research is required in this chapter and other lesser invasive protocols should be looked for prior to opting for the last option.
